# Using research priority-setting to guide bridging the implementation gap in countries – a case study of the Uganda newborn research priorities in the SDG era

**DOI:** 10.1186/s12961-019-0459-5

**Published:** 2019-05-31

**Authors:** Peter Waiswa, Monica Okuga, Lydia Kabwijamu, Joseph Akuze, Hanifah Sengendo, Patrick Aliganyira, Patricia Pirio, Claudia Hanson, Frank Kaharuza

**Affiliations:** 1 Department of Health Policy, Planning and Management, Makerere University School of Public Health, Kampala, Uganda; 2 Makerere University, Center of Excellence for Maternal, Newborn and Child Health, Kampala, Uganda; 3 Department of Public Health Sciences, Karolinska Institutet, Stockholm, Sweden; 4 Saving Newborn Lives, Save the Children, Kampala, Uganda; 5 London School of Hygiene and Tropical Medicine, London, United Kingdom

**Keywords:** Newborn research priorities, research agenda, setting priorities, Uganda

## Abstract

**Background:**

One of the greatest challenges that countries face regarding the achievement of the Sustainable Development Goal (SDG) targets for child health regard the actions required to improve neonatal health; these interventions have to be informed by evidence. In view of the persisting high numbers of newborn deaths in Uganda, we aimed to define a locally contextualised national research agenda for newborn health to guide national investments towards SDG targets.

**Methods:**

We adopted a systematic approach for priority-setting adapted from the Child Health and Nutrition Research Initiative. We identified and listed local newborn researchers and experts in Uganda by reviewing the PubMed database, through a snowballing technique, and engaged the Ministry of Health. Participants were requested to generate at least three research questions. The collated questions were sent to the same expert group to be rated using five criteria, including answerability, scalability, impact, generalisability and speed.

**Findings:**

Of the 300 researchers and stakeholders contacted, 104 responded (36%) and generated 304 questions. These questions were collated and duplicates removed giving a condensed list of 41 research questions. These questions were then rated by 82 experts. Of the top 15 research questions, 86.7% (13/15) were in the service delivery and 6.7% (1/15) in the development domain, while only 6.7% (1/15) was in the group ‘other’. None of the leading 15 questions was in the discovery domain. Strategies to improve quality of intrapartum care featured high in the responses, while research around care for premature babies was not a perceived focus of research.

**Conclusions:**

The focus of improved evidence to guide and innovate service delivery, foremost intrapartum care, reflects the importance of this area as accelerated improvement is likely to yield fast and sustained survival gains in the neonatal period and beyond in Uganda. We recommend that other countries adapt a similar approach in defining priority reproductive, maternal, newborn and child health areas for investment in order to accelerate progress towards achieving the SDGs.

**Electronic supplementary material:**

The online version of this article (10.1186/s12961-019-0459-5) contains supplementary material, which is available to authorized users.

## Introduction

Globally, newborn mortality remains high, with 2.7 million newborns dying each year [1]. Three-quarters of newborn deaths occur in the first week of life. The leading causes of mortality in this group are infections such as pneumonia and sepsis (36%), preterm birth complications (28%) and birth asphyxia (23%) [2, 3].

While the Millennium Development Goals had set the target of reducing child mortality by two-thirds, this target was missed and a reduction of only 49% was reported at the end of this period, with a comparatively smaller reduction in neonatal mortality being observed [4]. In response, new ambitious Sustainable Development Goal (SDG) targets for reducing newborn deaths to not more than 12 per 1000 live births in all countries by 2030 have been set [5]. These ambitious targets, embracing health, growth, development and survival, call for concerted and intensified efforts to prevent and address the underlying conditions to tackle the ‘unfinished business’ of the Millennium Development Goal era.

To support the ambitious targets, global research priorities have been identified, including innovative approaches and platforms to deliver known effective interventions [6, 7] aimed at providing the missing evidence required to reduce neonatal mortality, morbidity and long-term impairment [8]. While it is important that these global research priorities are formulated to guide investments, national priority-setting should complement such international guidance to be most relevant for the specific health system context and epidemiological situation. Moreover, there is a dire need to bridge the improvement–science–gap, as raised by Marshall et al. [9], where silo thinking within science (and academia) and health service and health sector development precludes the implementation and rigorous evaluation of programmes aiming to improve care. Few projects evaluating implementation strategies have been rigorously tested, and those who have been, have generated questions rather than providing answers; for example, the large Safe Childbirth Checklist trial – assessing the effect of the implementation of a checklist during labour combined with coaching – indicated that the intervention had no effect on mortality [10]. The authors concluded that aspects of the context and resource constraints as well as lack of referral facilities might have diminished any effect. In addition, the contribution of the knowledge and professionalisation of the health providers in these facilities to the lack of any effect will need to be reviewed. This is one of many proposed theories as to why the checklists did not have impact. Another strategy that has received increasing attention in low-resource settings is quality improvement and quality management. Again, the evidence of the effect of these is limited. However, promising effects have been reported where human resources and supplies were not limiting factors for implementation [11–13], thus posing questions regarding the thresholds wherein approaches can bear fruit in health systems in low- and middle-income countries.

Uganda made significant progress in reducing child deaths during the Millennium Development Goal era. Child mortality reduced from an estimated 148/1000 live births in 2000 to 55/1000 in 2015, representing a 4.9% annual reduction rate [14]. However, the latest results from the recent Uganda Demographic Health Survey 2016 indicate that neonatal mortality has stagnated at 27 deaths per 1000 live births over the last decade [15]. Global estimates propose that, currently, 39,000 newborns die in Uganda per year, and another 40,000 are born as stillbirths. These babies continue to die mainly from infections, prematurity and birth asphyxia, similar to the causes of deaths in most low- and middle-income countries.

Uganda has made key commitments summarised in the Reproductive Maternal Newborn Child and Adolescent Health sharpened plan [16] and has also signed into the Every Newborn Action Plan commitments and targets [6]. The 5-year investment plan anchored in the Health Sector Development Plan targets for 2020 [17] aims at reducing neonatal mortality from 27 to 16 per 1000 live births. In order to achieve this, relevant locally driven research is needed to guide the limited research capacity and funding to obtain maximum impact on newborn health. Ugandan policy-makers and health workers need sufficient evidence to plan for appropriate, cost effective and contextually relevant models for newborn care. Research is needed to understand what has been done and to prioritise what needs to be done in Uganda so as to achieve the SDG targets. With the required evidence at hand, the country can then innovate and intensify efforts to reduce neonatal mortality and attain the set SDG targets. This context-specific research agenda will provide the much need direction and guidance to reduce the knowledge gap to drive the reduction in neonatal morbidity and mortality in Uganda.

## Methods

**Fig. 1:**
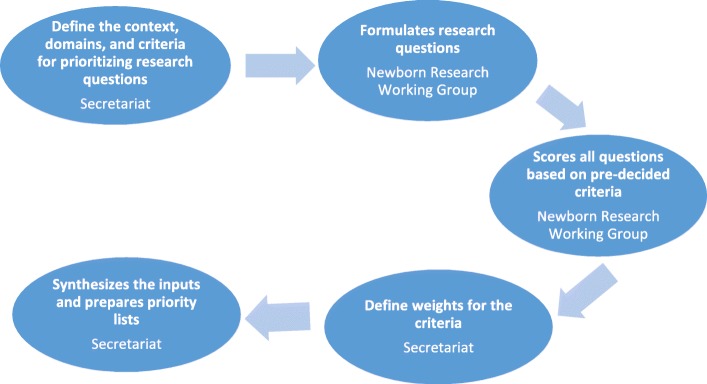
Summary of the adapted Child Health and Nutrition Research Initiative methodology. Adapted from the preterm research agenda [22]

We adopted a systematic approach for priority-setting adapted from the Child Health and Nutrition Research Initiative (CHNRI) methodology for research agenda-setting similar to the methods used to define the global newborn research agenda [8] and in previous priority-setting exercises by WHO on the five major causes of child deaths [18–22]. In brief, CHNRI is a four-stage process. The first stage is to understand the context under study, collecting ideas from stakeholders, setting up domains for categorisation of the ideas and establishing criteria for prioritising the research questions. The next stage is to formulate research questions from the ideas. The third stage involves getting the stakeholders to score all the research questions on the pre-established criteria. Lastly, the received questions are scored using pre-determined scoring criteria and the questions ranked (Fig. 1).

We identified and listed local newborn researchers in Uganda through reviewing the PubMed database and through a snowballing technique. We further engaged the Ministry of Health in identifying the most active newborn programme experts in Uganda. Each of the identified experts was requested to generate at least three research questions that, if answered, could lead to improvement of newborn health outcomes in Uganda from 2016 to 2021. The researchers received a questionnaire through the Survey Monkey platform (Additional file 3). For a period of 8 weeks, the 300 identified stakeholders/experts received, on a weekly basis, an email reminder concerning the questions/ideas requested for. These questions generated were collated and duplicates removed and refined by a small group of six maternal and newborn experts from the Makerere University Centre of Excellence for Maternal, Newborn and Child health. The response rate and number of questions collated are reported in the results section.

### Defining context, domains and criteria for prioritisation

The secretariat at the Makerere University Centre of Excellence for Maternal, Newborn and Child Health defined the scope of research to include any determinant that can influence newborn health and have an impact on mortality. With this in mind, the proposed research questions were refined based on the Population, Intervention, Comparison and Outcome (PICO) format [23] and further reduced. These research questions were then categorised into four domains (Table 1) adopted from the research pipeline and used to set the global agenda [8]. Criteria for scoring and thus prioritisation of the questions was adapted from criteria used in setting the global newborn research priorities [8]. This included scoring the questions against a five-point criteria, which included (1) answerability – the likelihood that the research question can be answered robustly and ethically; (2) scalability – the likelihood that the new knowledge can be applied to neonatal programmes at scale; (3) impact – the likelihood that the new knowledge will lead to programme changes that will increase survival and reduce morbidity of newborns; (4) generalisability – the likelihood that the new knowledge can be applied in diverse, high-need geographies; and (5) speed – the likelihood that the new knowledge can be generated in 1–5 years (Additional file 1).

**Table 1:** Domains in the research pipeline. Adapted from Yoshida et al. [8]

Domain	Description
Discovery	Research aimed at finding new solutions such as new medicines, vaccines or other preventive interventions, or new diagnostics
Development	Research aimed at improving existing interventions, reducing their costs or making them simpler to deliver
Service Delivery	Research that would help deliver existing interventions to more mothers and newborns with high quality
Other	Anything that did not fit in the first three domains

For each question, each criterion was scored as either 1, 0 or 0.5, where 1 represented a yes, 0 represented no or do not know, while 0.5 represented possibly.

### Formulating questions

A stakeholders’ meeting was held to review and refine the submitted questions. The stakeholders engaged were chosen from the original list of 300 researchers on the basis of availability at that particular time. The questions were edited, duplicates removed and some questions re-worded. The final list of questions was programmed into a Microsoft (MS) Excel form.

### Scoring questions using pre-defined weights

The final list of 41 questions was sent out for scoring to the 300 stakeholders who had originally been identified. This list was sent via webmail as an MS Excel form or as paper-based tables via post or hand delivery. Respondents were requested to score each of the questions against a predetermined five-point score criteria, including answerability, scalability, impact, generalisability and speed of generating the required information.

### Data management and reconciliation

The completed MS Excel forms and the paper-based tables were returned to the secretariat. The paper-based tables were entered into a pre-programmed MS Excel data entry form. The data in the MS Excel forms was combined and reconciled using MS Excel-based Visual Basic macros and programming.

### Analysis and synthesis of results

The secretariat analysed the responses using Microsoft Excel (2013). The sum scores for each criterion for each question were computed. The average score for each criterion of each question was computed as the sum of scores for that particular criterion divided by the total number of scorers. The Research Priority Score for each question was then calculated as the average score of each of the criteria divided by total number of criteria and expressed as a percentage, as follows: Research Priority Score = (average Criterion 1 score + average Criterion 2 score + average Criterion 3 score + average Criterion 4 score + average Criterion 5 score)/5.

## Results

**Fig. 2:**
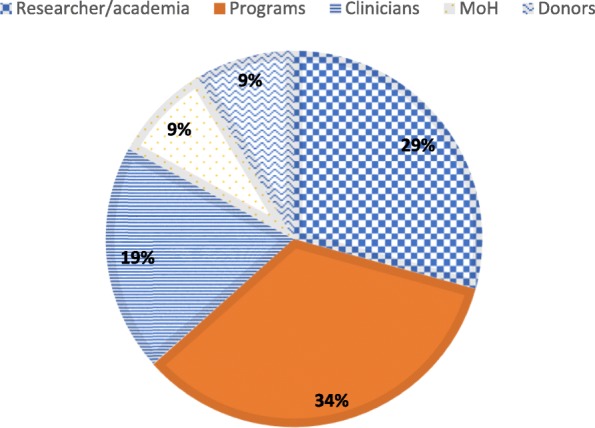
Secretariat and stakeholder processing from generation to scoring of questions

**Fig. 3:**
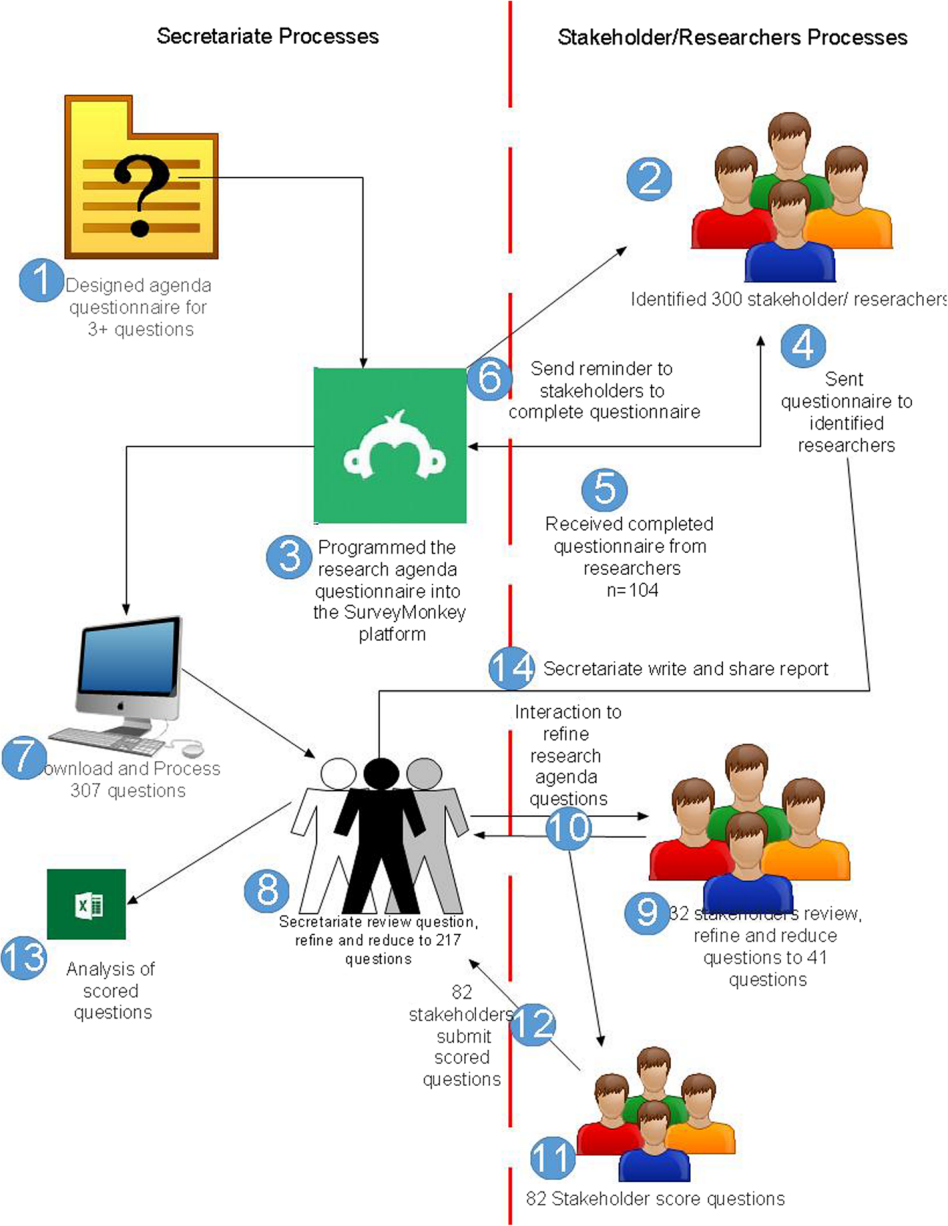
Experts who scored the questions by category

Of the 300 maternal and newborn health (MNH) researchers and stakeholders contacted, 104 responded (36.0%). Of the 104 researchers (100% submitted at least one question, 95% submitted at least two questions, 87% submitted at least three questions and < 36% of respondents submitted four or more questions), 307 questions were generated. These questions were reduced in a two-step process, with the first step involving only the secretariat and then second step involving stakeholders during a stakeholder meeting. The 307 questions received by the secretariat were reviewed, refined, grouped into the four domains and reduced to 217 questions (Additional file 2). Furthermore, the 217 questions were reviewed and reduced by the 32 stakeholders to formulate 41 final research agenda questions (Figs. 2 and 3).

### Sociodemographic characteristics of the respondents

The 41 questions were scored by 82 of the 300 experts contacted, giving a response rate of 27.3%. The majority (94.0%) of respondents were Ugandan by nationality, whereas 6.0% were foreigners. Approximately 34.0% of the scorers were programme personnel in MNH, 29.2% researchers, 19.5% clinicians, 8.5% Ministry of Health personnel/policy-makers and 8.5% donors (Fig. 3).

### Research Priority Scores

The overall percentage research priority scores for the 41 scored questions ranged from 27.2% to 80.6%. Of the top 15 research questions, 86.7% (13/15) were in the service delivery domain while 6.7% (1/15) were development and only 6.7% (1/15) was in the group termed ‘other’, which was comprised of epidemiological questions, socioeconomical suggestions, etc. (Table 2). None of the leading 15 questions was in the discovery domain.

**Table 2:** Top 15 Uganda newborn research agenda priority-setting questions

	Question	Domain	Percentage Research Priority Score (%)	Answerability	Scalability	Impact	Generalizability	Speed
1	How can we effectively sustain partograph use for labour management?	Delivery	80.6	81.1	80.5	80.5	80.4	80.5
2	Can participatory/women groups improve neonatal health in the Ugandan setting?	Delivery	80.5	83.5	80.5	79.2	79.2	79.9
3	How can we effectively maintain clinical competencies for newborn care in health facilities?	Delivery	79.9	81.7	80.5	79.9	79.2	78.1
4	How can we improve newborn outcomes among vulnerable populations?	Delivery	79.6	81.71	81.10	79.3	78.7	77.4
5	What low cost technologies improve neonatal survival in community and facility setting in Uganda?	Development	79.3	81.7	79.3	78.7	78.7	78.1
6	What is the aetiology of stillbirths in Uganda?	Other	77.7	78.1	78.1	78.1	78.1	76.2
7	Does knowledge of essential newborn care practices among mothers have an impact on newborn survival?	Delivery	74.9	83.5	82.3	81.1	82.3	45.1
8	How can male involvement be used to improve neonatal outcomes?	Delivery	74.3	82.9	81.1	82.3	79.9	45.1
9	Can integration of essential neonatal care into lower health facilities improve neonatal outcomes?	Delivery	74.1	83.5	80.5	81.1	81.1	44.5
10	How can newborn referral and follow-up be improved at community and facility level?	Delivery	73.7	82.3	80.5	80.5	81.1	43.9
11	Can involvement of newborn champions in the political, social and economic arena improve newborn outcomes?	Delivery	73.6	82.9	45.1	80.5	80.5	78.7
12	What is the level of skills of midwives in neonatal resuscitation in Uganda?	Delivery	73.5	46.3	81.1	79.9	79.9	80.5
13	Can the use of simple algorithms by community health workers to identify and refer neonates with danger signs improve neonatal outcomes?	Delivery	73.4	82.9	79.9	80.5	80.5	43.3
14	Can integration of culturally relevant practices within maternal and newborn care improve uptake of institutional deliveries?	Delivery	73.0	82.9	80.5	79.9	79.9	42.1
15	What is the feasibility of improving access to neonatal sepsis management using simplified antibiotics for newborns when referral to hospital is not possible?	Delivery	72.8	81.7	80.5	79.3	79.3	43.3

The leading questions in the delivery domain focused on effective use of partographs, improvement in health worker skills and use of models such as peer groups to improve care. Of the top 15 questions, nine (60.0%) were focused on integrated care, while four (26.7%) addressed intrapartum care. Only two (13.3%) of the questions focused on newborn sepsis.

Of the 41 questions, three (7.3%) emerged in the ‘development’ domain and these ranked between the 4th and 37th position using the research priority score ranking (Table 3). Ideas in this domain included improving care in vulnerable populations, low cost technologies that enhance newborn survival, improving infection diagnosis by community health workers (CHWs) and postpartum care. All the priorities in this domain addressed general care along the continuum.

**Table 3:** Questions in the development domain

Rank	Question	Domain	Percentage Research Priority Score (%)
5	What low cost technologies improve neonatal survival in community and facility setting in Uganda?	Development	79.3
33	What models of postpartum care can be used to improve newborn care practices and reduce mortality?	Development	50.7
37	What is the feasibility of use of low-cost technologies in gestation age dating in Uganda?	Development	48.4

**Table 4:** Questions in the ‘other’ domain

Rank	Question	Domain	Percentage Research Priority Score (%)
6	What is the aetiology of stillbirths in Uganda?	Other	77.6
20	How can we sustainably align partner and government support for better maternal and neonatal outcomes?	Other	66.4
21	What are the major social economic determinants for newborn survival?	Other	65.8
25	How can community structures and practices be harnessed to improve maternal and neonatal survival?	Other	63.9
27	How can Public Private Partnership model be fully enhanced to improve newborn health?	Other	59.7
28	Can social marketing improve male involvement in maternal programmes?	Other	58.5
29	What is the contribution of caesarean section to maternal and newborn morbidity and mortality?	Other	58.4
30	What are the barriers to translating evidence-based knowledge in maternal, newborn and child health into action in various localities in Uganda?	Other	58.2
36	What pathogens dominate the neonatal infections in Uganda?	Other	50.3
40	How much more investment (resources) do we need to reduce newborn mortality by half?	Other	35.4
41	What biomarkers to predict preterm birth can be developed for scale up in Uganda?	Discovery	27.1

The domain ‘other’, which was comprised of questions that could neither fit in the delivery nor the development domains, had a total of 11 out of the 41 questions (26.8%). These ranked between 6th and 41st of all the research questions and included epidemiological, research, and socioeconomic questions, amongst others (Table 4). Five of these priorities had an epidemiological aspect and included aetiology of stillbirths, socioeconomic determinants for newborn survival, prevalence of mortality due to caesarean sections, causative pathogens for neonatal sepsis, and biomarkers to predict preterm labour.

## Discussion

Uganda represents the first country in the global South to systematically conduct and publish a country-specific newborn research agenda to complement those developed globally. As expected, the Uganda newborn research priorities focus largely on the delivery of newborn care services followed by development aspects. Development research questions were only found to rank 5th, 33rd and 37^th^, while discovery research was ranked at a distant number 41.

Eight (80.0%) of the top ten priorities and 65.8% (27/41) of all identified research priorities were found in the delivery category. This is not surprising given the large number of preventable deaths that can be averted with well-established, simple and cost-effective solutions if all mothers and babies would be reached [7]. Investing in delivery solutions will also yield the greatest return in the short term and can be translated more quickly into measurable and equitable benefits in terms of morbidity and mortality reduction. Achieving this requires a close interface between researchers, implementers and policy-makers so that issues of funding, designing, implementation and use of findings are addressed [9]. Implementation science will need to be at the heart to establish what works for whom, why and in which context [24, 25]. Our analysis clearly supports this dire need to find ways to develop strategies to support the implementation of evidence-based guidelines in low-resource countries as highlighted also by the Commission on High Quality Health Systems in the SDG area [26]. The first priority research questions under the delivery domain relate to finding ‘facilitators’ that are aligned to the context and support what ought to be implemented according to local guidelines, in a way that health providers feel empowered as, for example, a facilitation intervention for antenatal care in Tanzania [27]. Maintaining clinical competences is another important aspect raised. More implementation science is probably needed if the presently promoted competency-based training is to be able to overcome challenges [28].

It is noted that the importance of implementation science questions have also been highlighted at the global level, for example, the global newborn research agenda and other disease-specific research agendas where most priorities are largely in the delivery domain [8, 19, 21, 22]. Further, the strong focus on delivery solutions might have been the result of engaging the Ministry of Health and implementation partners.

Cross-cutting similarities between our findings and those in the global newborn research priorities include improving the quality of care around the time of birth, implementation of interventions at primary level facilities, addressing access barriers, and improvement in skills of CHWs, including improving access to neonatal sepsis management using simplified antibiotics for newborns when referral to hospital is not possible. The strong focus on improving service delivery in primary facilities and the community level should be viewed against the background that, in Uganda, CHWs participate in treatment of older children under Integrated Community Case Management [29, 30], while they are, by policy, not allowed to treat neonates. They are only mandated to conduct health promotion and facilitate referrals [31–34]. In other settings, such as Bangladesh, CHWs are allowed to treat neonatal sepsis with injectable antibiotics and thus have led to a substantial reduction in neonatal mortality [35].

Globally, there seems to be a shift in focus from CHWs to improving the quality of facility-based care. Further synthesis of our findings reveals that improving the quality of perinatal care is receiving more attention. This is consistent with current global trends in which quality of care is increasingly recognised as a critical aspect of the unfinished maternal, newborn and child health agenda [36, 37]. Our results are also consistent with the newly released WHO guidelines on quality improvement for MNH and the guidelines on care at birth and in pregnancy [38, 39]. Furthermore, Uganda is one of the pioneer countries in the WHO Quality of Care Network, which aims to improve the quality of care provided [37]. A number of studies have revealed a low quality of care provided in many health facilities in Uganda [40, 41]. This research agenda can therefore guide areas of concern as the Ministry of Health seeks to coordinate quality improvement approaches countrywide.

The major themes in the ‘development’ domain included developing models for better postnatal care, adapting low cost technologies for gestation age estimation as well as improving neonatal survival in community and facility settings generally. In view that the most frequent cause of neonatal mortality in Uganda, as published by international researchers, is prematurity followed by neonatal infections and asphyxia [42], we would have expected more explicit ideas on how we can improve survival of preterms and successfully manage infections and asphyxia. It is surprising to us that interventions like Kangaroo Mother Care were not specifically mentioned as priorities. This is in contrast to the global research priorities where, in this domain, six of the top ten priorities were identified in the areas of preterm birth, intrapartum-related events and newborn infections [8].

Our research priority-setting led to only one research question in the discovery domain ‘biomarkers for prediction of preterm birth’ and a few which could not be classified and were thus in the ‘other’ domain. These included epidemiological studies on the aetiology of stillbirths, managing and coordination of development partners, and investments required to reduce mortality, enhancing public private partnerships and social marketing for better newborn health.

The low priority of ‘discovery’ and ‘development’ domains is consistent with findings from other studies, probably because themes from these domains take a longer duration to be translated to quantifiable benefits such as reduction in mortality and therefore are not a common area of choice among experts [8, 21, 22]. However, these domains still remain important in developing innovations and evidence that may be fed into the continuum of the research pipeline [43]. On the other hand, it may be logical for low-income countries such as Uganda to achieve mortality reduction using the known feasible interventions prior to exploring development of other models or strategies.

Notwithstanding, the Uganda newborn research agenda is not a standalone document, it is anchored and aligned with Uganda’s key policy documents, for example, the National Newborn Health Advocacy Strategy (2014–2018), the Investment Case for Reproductive Maternal, Newborn, Child and Adolescent Health (2016–2020) [16] and the Health Sector Development Plan (2015/2016–2020) [17]. All these policy documents have one focus of accelerated progress towards reduction of maternal, newborn and child mortality in Uganda. Even though there are some significant overlaps with the global newborn research agenda, for example, in quality of intrapartum care, improving illness diagnosis and treatment of sepsis by CHWs at community level, most of the questions are context specific with regards to Uganda. This is especially because they were developed by experts in Uganda who are aware of the Ugandan context. This shows the value of globally developed research priorities being followed by local adaptation, as otherwise they may not be relevant.

Our study’s strength was that it is a locally driven process with participation not only of researchers, but also policy-makers and programme implementers, including the Ministry of Health. We think this will increase its acceptability and relevance, and perhaps future investment. Indeed, to our knowledge, this is the first country-specific research agenda to be developed in a systematic and participatory way in the SDG era. However, there are also some limitations to this study. First, there was a low response rate of 36%; despite this being better than the response rate of 22% for the global newborn agenda [8], it could raise questions as to whether this is a true representation of the newborn experts in Uganda. Secondly, there are limitations with the CHNRI methodology used. The results may reflect the thoughts and interests of the experts rather than the current research needs in Uganda. However, we tried to mitigate these limitations by working together with the Ministry of Health to identify a wide range of newborn experts within the country. Conversely, the strengths of this methodology include transparency and the high level of agreement between experts on the research priorities.

## Conclusion

In this era of the SDGs, we need to re-think and innovate service delivery if we are to achieve country-specific MNH targets. It is therefore critical to prioritise the research we need to undertake to reap the most benefits but with consideration of the cost implications and in consultation between implementers and researchers. The results presented here imply that more attention should be given to the delivery mechanisms and implementation science, especially around the time of birth, whilst simultaneously addressing barriers that hinder improved newborn outcomes. Research done in the suggested areas could provide the necessary evidence to address the key gaps in knowledge needed to reduce newborn mortality substantially in Uganda. To achieve this, we call for funding of implementation research so that policy-makers and programme managers can work together with academia in finding effective solutions to local causes of morbidity and mortality, and in solving service delivery barriers of maternal, newborn and child health.

## Additional files

Additional file 1: Scoring criteria for questions. (XLSX 21 kb)

Additional file 2: Original and refined questions. (XLSX 31 kb)

Additional file 3: Survey monkey. (DOCX 163 kb)

## Data Availability

The datasets generated and analysed during this study are available on request from the corresponding author.

## Abbreviations

CHNRI: Child Health and Nutrition Research Initiative
CHWs: community health workers
MNH: Maternal and Newborn Health
SDGs: Sustainable Development Goals

## References

[1] You D, Hug L, Ejdemyr S, Beise J, Perez-Escamilla R, Moran V (2016). Levels and trends in child mortality. Estimates developed by the UN Inter-agency Group for Child Mortality Estimation (IGME). Report 2015. Matern Child Nutr.

[2] Liu L, Johnson HL, Cousens S, Perin J, Scott S, Lawn JE (2012). Global, regional, and national causes of child mortality: an updated systematic analysis for 2010 with time trends since 2000. Lancet.

[3] Bhutta ZA, Chopra M, Axelson H, Berman P, Boerma T, Bryce J (2010). Countdown to 2015 decade report (2000–10): taking stock of maternal, newborn, and child survival. Lancet.

[4] Liu L, Hill K, Oza S, Hogan D, Chu Y, Cousens S, Black RE, Laxminarayan R, Temmerman M (2016). Levels and causes of mortality under age five years. Reproductive, Maternal, Newborn, and Child Health.

[5] World Health Organization. Health in (2015). from MDGs to SDGs. Report.

[6] World Health Organization (2014). Every Newborn: An Action Plan to end Preventable Deaths.

[7] Lawn JE, Blencowe H, Oza S, You D, Lee AC, Waiswa P (2014). Every Newborn: progress, priorities, and potential beyond survival. Lancet.

[8] Yoshida S, Martines J, Lawn JE, Wall S, Souza JP, Rudan I (2016). Setting research priorities to improve global newborn health and prevent stillbirths by 2025. J Glob Health.

[9] Marshall M, Mountford J (2013). Developing a science of improvement. J R Soc Med.

[10] Semrau KEA, Hirschhorn LR, Marx Delaney M, Singh VP, Saurastri R, Sharma N (2017). Outcomes of a coaching-based WHO safe childbirth checklist program in India. N Engl J Med.

[11] World Health Organization. Quality, Equity, Dignity: Improving Quality of Care to Achieve Ambitious SDG Targets to End Preventable Maternal, Newborn and Child Deaths. Geneva: WHO. http://www.who.int/pmnch/media/news/2017/improving-qualitycare-achieve-sdgtargets.pdf. Accessed 15 Apr 2018.

[12] Waiswa P, Manzi F, Mbaruku G, Rowe AK, Marx M, Tomson G (2017). Effects of the EQUIP quasi-experimental study testing a collaborative quality improvement approach for maternal and newborn health care in Tanzania and Uganda. Implement Sci.

[13] Twum-Danso NA, Dasoberi IN, Amenga-Etego IA, Adondiwo A, Kanyoke E, Boadu RO (2014). Using quality improvement methods to test and scale up a new national policy on early post-natal care in Ghana. Health Policy Plan.

[14] You D, Hug L, Ejdemyr S, Beise J (2015). Levels and Trends in Child Mortality. Report 2015. Estimates Developed by the UN Inter-Agency Group for Child Mortality Estimation.

[15] Uganda Bureau of Statistics (2016). Uganda Demographic and Health Survey 2016.

[16] Minisitry of Health (2016). Investment Case. Reproductive, Maternal, Newbornn, Child and Adolescent Health Sharpend Plan for Uganda.

[17] Ministry of Health (2015). Health Sector Development Plan 2015/16–2019/20.

[18] Bahl R, Martines J, Ali N, Bhan MK, Carlo W, Chan KY (2009). Research priorities to reduce global mortality from newborn infections by 2015. Pediatr Infect Dis J.

[19] Rudan I, El Arifeen S, Bhutta ZA, Black RE, Brooks A, Chan KY (2011). Setting research priorities to reduce global mortality from childhood pneumonia by 2015. PLoS Med.

[20] Fontaine O, Kosek M, Bhatnagar S, Boschi-Pinto C, Chan KY, Duggan C (2009). Setting research priorities to reduce global mortality from childhood diarrhoea by 2015. PLoS Med.

[21] Lawn JE, Bahl R, Bergstrom S, Bhutta ZA, Darmstadt GL, Ellis M (2011). Setting research priorities to reduce almost one million deaths from birth asphyxia by 2015. PLoS Med.

[22] Bahl R, Martines J, Bhandari N, Biloglav Z, Edmond K, Iyengar S (2012). Setting research priorities to reduce global mortality from preterm birth and low birth weight by 2015. J Glob Health.

[23] Santos CMC, Pimenta CAM, Nobre MRC (2007). The PICO strategy for the research question construction and evidence search. Rev Lat Am Enferm.

[24] Van Belle S, Rifkin S, Marchal B (2017). The challenge of complexity in evaluating health policies and programs: the case of women’s participatory groups to improve antenatal outcomes. BMC Health Serv Res.

[25] Marchal B, Kegels G, Van Belle S, Emmel N, Greenhalgh J, Manzano A, Monaghan M, Dalkin S (2018). Realist evaluation in health policy and systems research: theory incarnate. Doing Realist Research.

[26] Kruk ME, Gage AD, Arsenault C, Jordan K, Leslie HH, Roder-DeWan S (2018). High-quality health systems in the Sustainable Development Goals era: time for a revolution. Lancet Global Health.

[27] Pallangyo E, Mbekenga C, Olsson P, Eriksson L, Bergström A (2018). Implementation of a facilitation intervention to improve postpartum care in a low-resource suburb of Dar es Salaam, Tanzania. Implement Sci.

[28] Ersdal HL, Singhal N, Msemo G, Kc A, Data S, Moyo NT (2017). Successful implementation of Helping Babies Survive and Helping Mothers Survive programs—An Utstein formula for newborn and maternal survival. PLoS One.

[29] Wanduru P, Tetui M, Tuhebwe D, Ediau M, Okuga M, Nalwadda C (2016). The performance of community health workers in the management of multiple childhood infectious diseases in Lira, northern Uganda–a mixed methods cross-sectional study. Glob Health Action.

[30] Kalyango JN, Rutebemberwa E, Alfven T, Ssali S, Peterson S, Karamagi C (2012). Performance of community health workers under integrated community case management of childhood illnesses in eastern Uganda. Malar J.

[31] Okuga M, Kemigisa M, Namutamba S, Namazzi G, Waiswa P (2015). Engaging community health workers in maternal and newborn care in eastern Uganda. Glob Health Action.

[32] Timsˇa L, Marrone G, Ekirapa E, Waiswa P (2015). Strategies for helping families prepare for birth: experiences from eastern central Uganda. Glob Health Action.

[33] Nalwadda CK, Waiswa P, Kiguli J, Namazzi G, Namutamba S, Tomson G (2013). High compliance with newborn community-to-facility referral in eastern Uganda: an opportunity to improve newborn survival. PLoS One.

[34] Kayemba CN, Sengendo HN, Ssekitooleko J, Kerber K, Källander K, Waiswa P (2012). Introduction of newborn care within integrated community case management in Uganda. Am J Trop Med Hyg.

[35] Baqui AH, Arifeen SE, Williams EK, Ahmed S, Mannan I, Rahman SM (2009). Effectiveness of home-based management of newborn infections by community health workers in rural Bangladesh. Pediatr Infect Dis J.

[36] Van den Broek N, Graham W (2009). Quality of care for maternal and newborn health: the neglected agenda. BJOG.

[37] World Health Organization. Quality of Care Network 2016. http://www.who.int/maternal_child_adolescent/topics/quality-of-care/network/en/. Accessed 15 Mar 2017.

[38] World Health Organization (2016). Standards for Improving Quality of Maternal and Newborn Care in Health Facilities.

[39] World Health Organization (2003). Pregnancy, Childbirth, Postpartum, and Newborn Care: A Guide for Essential Practice. Geneva: WHO.

[40] Tetui M, Ekirapa EK, Bua J, Mutebi A (2012). Quality of antenatal care services in eastern Uganda: implications for interventions. Pan African Med J.

[41] Waiswa P, Akuze J, Peterson S, Kerber K, Tetui M, Forsberg BC (2014). Differences in essential newborn care at birth between private and public health facilities in eastern Uganda. Glob Health Action.

[42] Waiswa P, Kallander K, Peterson S, Tomson G, Pariyo GW (2010). Using the three delays model to understand why newborn babies die in eastern Uganda. Tropical Med Int Health.

[43] Moran M, Guzman J, Ropars A-L, McDonald A, Jameson N, Omune B (2009). Neglected disease research and development: how much are we really spending. PLoS Med.

