# Supplementary figures and images for: Using research priority-setting to guide bridging the implementation gap in countries – a case study of the Uganda newborn research priorities in the SDG era
Source: Health Res Policy Syst. 2019 May 31;17:54. doi: 10.1186/s12961-019-0459-5 (PMC6544968; doi:10.1186/s12961-019-0459-5)

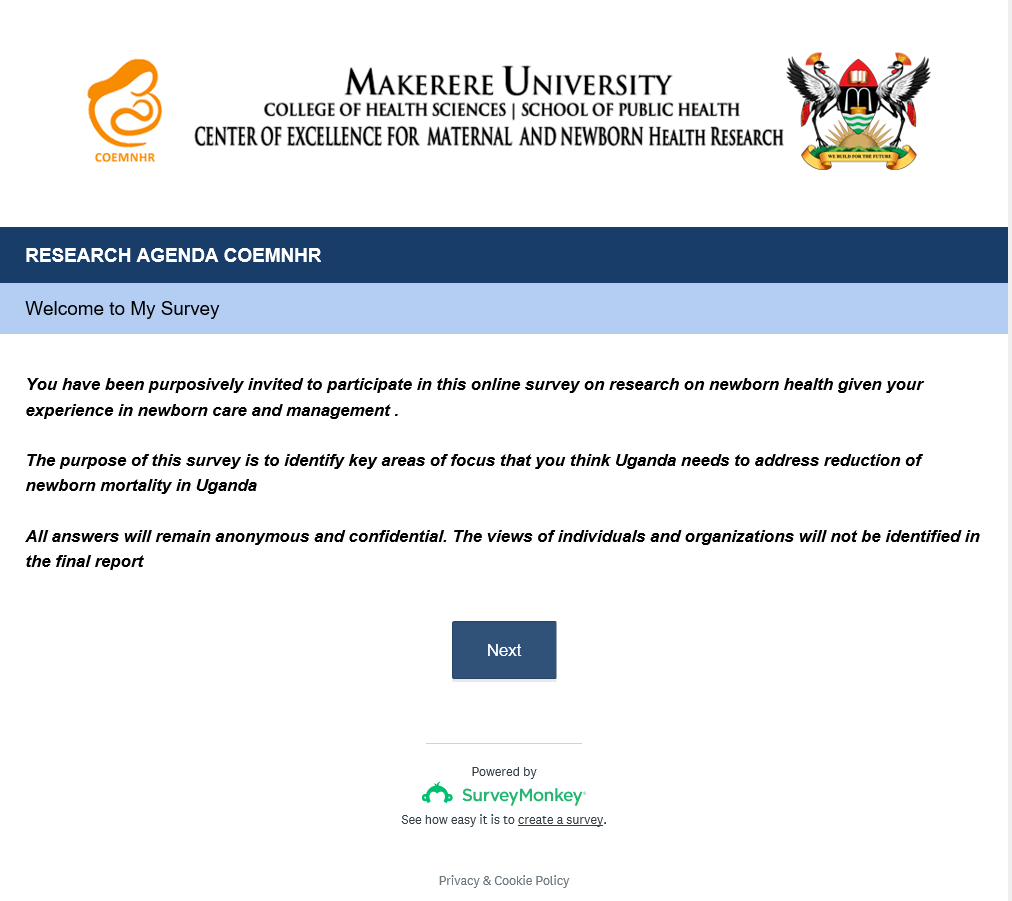


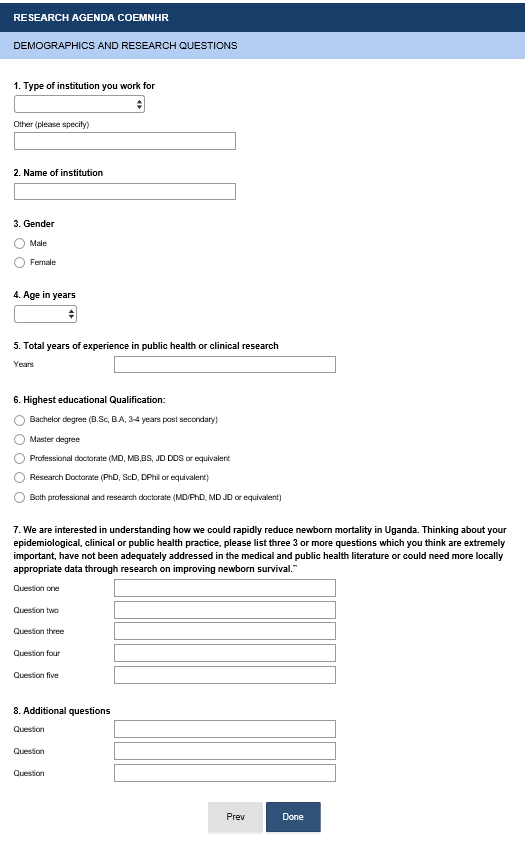

Supplement: Supplementary file 3 — Survey monkey. (DOCX 163 kb) [file 12961_2019_459_MOESM3_ESM.docx]
